# Water use efficiency and evapotranspiration in maize-soybean relay strip intercrop systems as affected by planting geometries

**DOI:** 10.1371/journal.pone.0178332

**Published:** 2017-06-09

**Authors:** Tanzeelur Rahman, Xin Liu, Sajad Hussain, Shoaib Ahmed, Guopeng Chen, Feng Yang, Lilian Chen, Junbo Du, Weiguo Liu, Wenyu Yang

**Affiliations:** 1 College of Agronomy, Sichuan Agricultural University, Chengdu, China; 2 Key Laboratory of Crop Ecophysiology and Farming Systems in the Southwest, Ministry of Agriculture, Chengdu, China; 3 College of Landscape Architecture, Sichuan Agricultural University, Chengdu, China; Zhejiang University, CHINA

## Abstract

Optimum planting geometries have been shown to increase crop yields in maize-soybean intercrop systems. However, little is known about whether changes in planting geometry improve the seasonal water use of maize and soybean intercrops. We conducted two different field experiments in 2013 and 2014 to investigate the effects of changes in planting geometry on water use efficiency (WUE) and evapotranspiration (ET_c_) of maize (*Zea mays* L.) and soybean [*Glycine max* (L.) Merr.] relay strip intercrop systems. Our results showed that the leaf area index of maize for both years where intercropping occurred was notably greater compared to sole maize, thus the soil water content (SWC), soil evaporation (E), and throughfall followed a decreasing trend in the following order: central row of maize strip (CRM) < adjacent row between maize and soybean strip (AR) < central row of soybean strip (CRS). When intercropped, the highest grain yield for maize and total yields were recorded for the 40:120 cm and 40:160 cm planting geometries using 160 cm and 200 cm bandwidth, respectively. By contrast, the highest grain yield of intercropped soybean was appeared for the 20:140 cm and 20:180 cm planting geometries. The largest land equivalent ratios were 1.62 for the 40:120 cm planting geometry and 1.79 for the 40:160 cm planting geometry, indicating that both intercropping strategies were advantageous. Changes in planting geometries did not show any significant effect on the ET_c_ of the maize and soybean intercrops. WUEs in the different planting geometries of intercrop systems were lower compared to sole cropping. However, the highest group WUEs of 23.06 and 26.21 kg ha^-1^ mm^-1^ for the 40:120 cm and 40:160 cm planting geometries, respectively, were 39% and 23% higher than those for sole cropping. Moreover, the highest water equivalent ratio values of 1.66 and 1.76 also appeared for the 40:120 cm and 40:160 cm planting geometries. We therefore suggest that an optimum planting geometry of 40:160 cm and bandwidth of 200 cm could be a viable planting pattern management method for attaining high group WUE in maize-soybean intercrop systems.

## Introduction

Scarce water resources is one of the crucial factors that contributes to the decline in agricultural productivity [1]. The current challenge in agriculture is to produce more yields by utilizing less water, especially in regions with limited land and water resources [2]. Maximizing crop water productivity by the most effective utilization of rain water resources is particularly important in arid land agriculture [3]. How scarce water resources can be better absorbed by crops, is the core objective of arid land agriculture [4].

Intercropping can improve crop yield by more efficient utilization of resources such as nutrient, water, and solar radiation [5–6]. Intercropping plays a pivotal role for increasing biodiversity, land use efficiency, nutrient and water use efficiencies, and enhanced ecological services [4, 7, 8]. Intercropping could be one of the potential ways to address some of the associated obstacles with modern agriculture, including low yield, pest and pathogen infection, soil degradation and environmental deterioration [9, 10], thereby promoting sustainable and productive agriculture [5].

Maize and soybean relay strip intercropping is practiced widely in southwest China by the application of three core technologies: varietal screening of maize and soybean plants, extending row spacing between maize and soybean strip, and inter-plant spacing reduction [7]. The land equivalent ratio in these modern mechanized based maize-soybean intercrop systems typically reached up to 2.27 and the corresponding average yields recorded were up to 11,475 kg ha^-1^ of maize and 2,364 kg ha^-1^ of soybean [11–13]. These yields were much higher than those produced by traditional maize-soybean intercropping [14–15].

Maize-soybean intercrop systems include a tall maize crop and a short soybean crop that grows as the subordinate late-sown crop of the pair [16]. The yield of soybean in maize-soybean relay intercrop systems has been reported to be lower because of the shading effect caused by maize on soybeans [17]. In our previous study, we found that an optimum planting geometry and bandwidth not only led to ameliorate the light environment of soybean canopy, but also boost the productivity of maize-soybean intercrop systems [13]. However, our understanding is limited about whether changes in planting geometry affect the WUE and ET_c_ of maize-soybean relay strip intercrop systems. A lot of studies have been previously reported on the WUE of intercrop systems, but with controversial results and many of them indicated that greater yields attained by the intercrops only as a consequence of higher water consumption [18–21]. The opportunities for increasing effective water use through intercropping are limited, especially in relay intercrop systems when the ground is sparsely occupied [9, 22]. Similarly, soil evaporation is the major component of evapotranspiration that contributes to the higher water consumption of agricultural systems, especially in relay intercrop systems due to incomplete ground cover [23–24]. However, little research has focused on the role of planting geometry in minimizing evaporative water loss of the relay strip intercrop systems.

We therefore arranged the present study to: (i) investigate the effects of changes in planting geometry on the canopy structure and coverage of maize plants; (ii) explore the relationships of soil water content, average maize LAI with maize narrow-row spacing, and soil evaporation, throughfall with average maize LAI; and (iii) determine an optimum planting geometry for attaining high WUE in maize-soybean relay strip intercrop systems.

## Materials and methods

### Ethics statement

All experiments were performed under the guidelines established by Sichuan Agricultural University, China. Approval of an ethics committee was therefore neither required nor obtained for the described field studies.

### Site description and crop management

**Fig 1:**
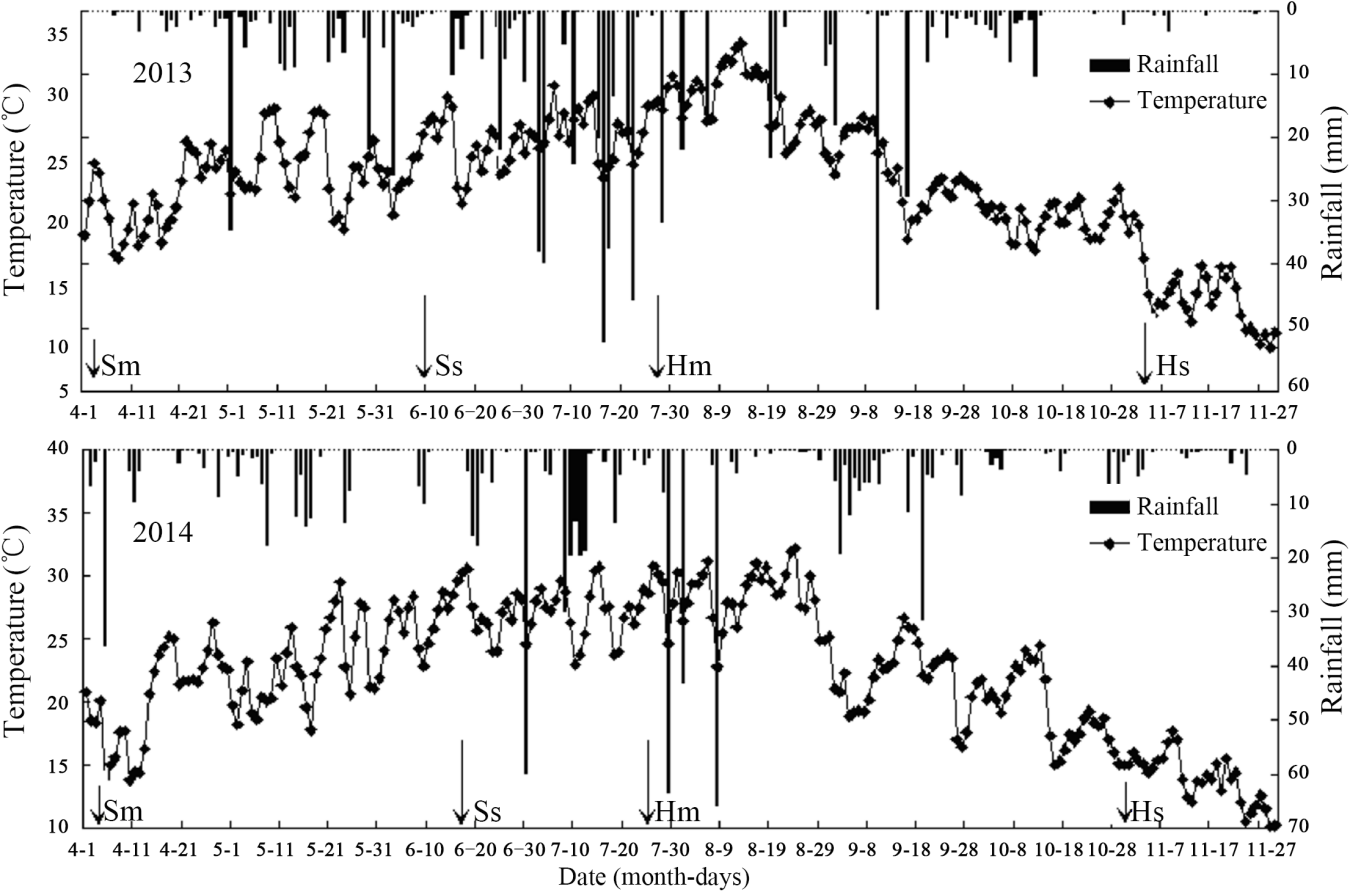
Mean air temperature and average rainfall of the experimental site in 2013 and 2014 seasons. The ‘Sm and Ss’ represent maize sowing and soybean sowing, whereas ‘Hm and Hs’ represent maize and soybean harvesting, respectively. Data were collected from the Renshou Bureau of Meteorology, Sichuan, China.

Two different field experiments were conducted at the research farm of Sichuan Agricultural University, Renshou county (30°4'16''N, 104°12'53''E), Sichuan province of China in 2013 and 2014 seasons. The study area is characterized by a sub-tropical, sub-humid type of climate. The soil class is sandy loam with a slope of about 12.3°. The soil bulk densities measured at 0–10 cm, 10–20 cm and 20–30 cm soil depths were 1.33, 1.34 and 1.52 g cm^-3^, respectively. The mean annual air temperature was 17.9°C and the average annual rainfall was 912 mm, which largely occurred in the co-growth period over June through August as shown in Fig 1.

The mean air temperature in 2013 was higher than that in 2014, whereas the average annual rainfall was lower in 2013 than that in 2014, resulting in higher relative humidities in 2014. Before seeding, N at 135 kg ha^-1^, P at 40 kg ha^-1^ and K at 10 kg ha^-1^ were applied to maize in both intercropped and sole cropped treatments, while soybeans in intercropping and sole cropping were fertilized with N at 75 kg ha^-1^, P at 40 kg ha^-1^ and K at 4 kg ha^-1^ in 2013 and 2014, respectively. Moreover, maize at (V6) and soybean at early bloom (R1) stage were also fertilized with N at 135 kg ha^-1^ and 75 kg ha^-1^, respectively.

### Experimental design

**Fig 2:**
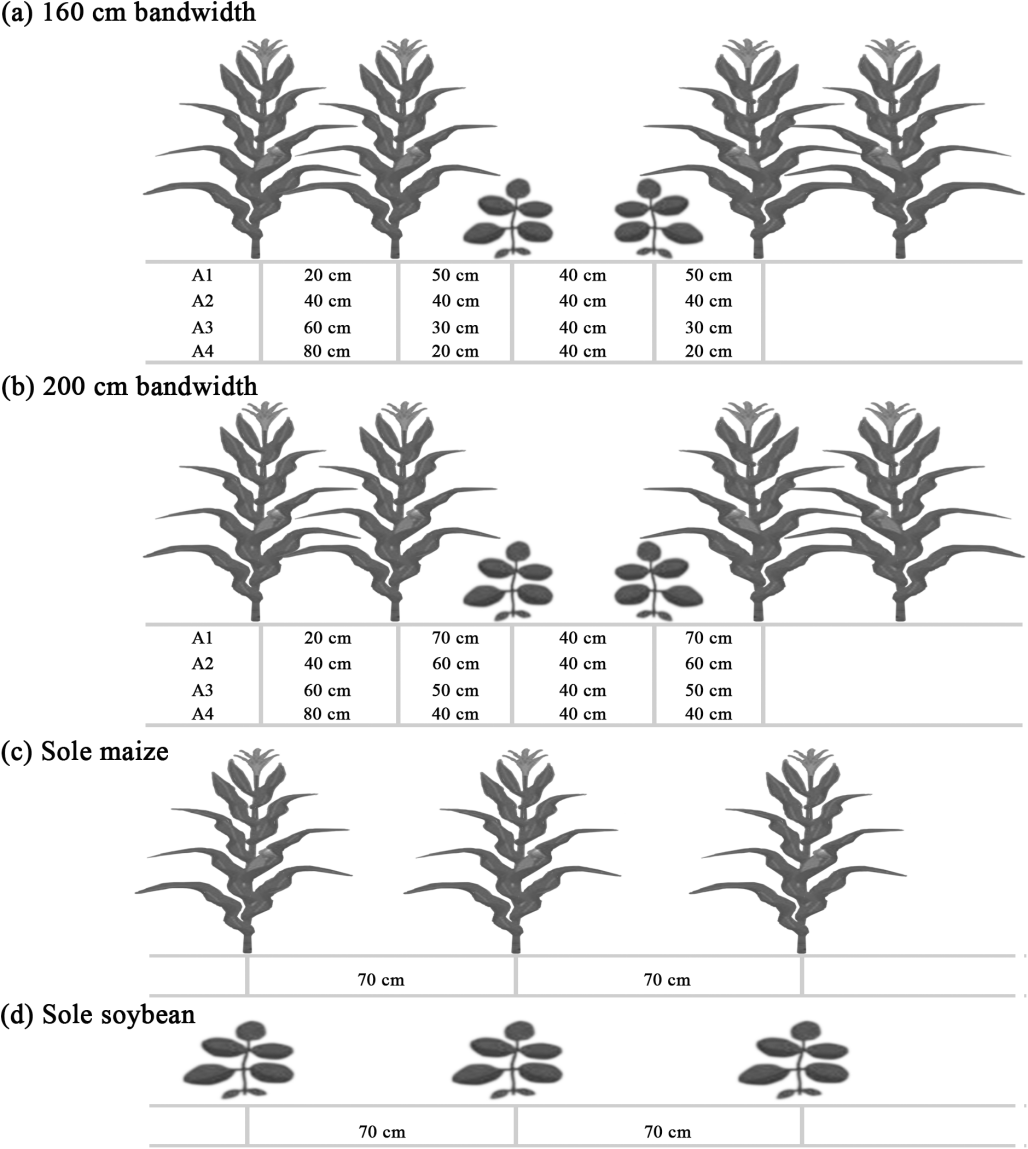
Row arrangments for maize-soybean relay strip intercrops and sole crops. (a) 160 cm bandwidth (b) 200 cm bandwidth (c) sole maize and (d) sole soybean. Two rows of maize were intercropped with two rows of soybean. Maize was planted in the narrow rows, whereas soybeans were planted in wide rows between the maize rows two months before maize maturity.

Two different field experiments of maize and soybean relay strip intercrops were designed by random complete block method with three replicates. Maize and soybean were planted as: intercropped maize-soybean (IMS), maize sole (M-sole), and soybean sole (S-sole). The selected maize and soybean cultivars were ‘‘Chuandan418” and ‘‘Nandou12”, respectively. In 2013, maize and soybean were intercropped in the following four different narrow-wide row planting patterns: ‘‘20:140 cm” (20 cm narrow maize row and 140 cm wide soybean row), ‘‘40:120 cm”, ‘‘60:100 cm”, and ‘‘80:80 cm” at 160 cm bandwidth, whereas in 2014 they comprised of ‘‘20:180 cm”, ‘‘40:160 cm”, ‘‘60:140 cm”, and ‘‘80:120 cm” at 200 cm bandwidth (Fig 2A–2D).

The row spacing of sole maize and sole soybean was kept constant at 70 cm. Each plot size was 24 m^2^ at 160 cm bandwidth and 30 m^2^ at 200 cm bandwidth. Maize and soybean were planted with 6 and 10 plants m^-2^, respectively, in both intercropped and sole cropped treatments. The plant spacing of intercropped maize was 20.9 cm and 16.7 cm and intercropped soybean was 12.5 cm and 10 cm at 160 and 200 cm bandwidth, respectively. Sole maize and sole soybeans were sown with 23.8 cm and 14.3 cm plant spacing, respectively. In 2013, the sowing dates were April 3 for maize and June 5 for soybean, whereas the dates of harvesting were July 28 for maize and November 4 for soybean. In 2014, the sowing dates were April 4 for maize and June 2 for soybean, while the dates of harvesting were July 26 for maize and October 31 for soybean.

### Measuring indices and methods

#### Dynamics of maize leaf area index

The LAI of maize plants was measured manually every 7–10 days from silking to physiological maturity in the three replicates of maize-soybean relay strip intercropping. For this purpose, five maize plants were destructively sampled from the sample area of each experimental plot. Samples from the boundary of maize-soybean intercrop systems were avoided. To determine leaf area, we measured the length and the greatest width of selected leaves with a ruler. Then the area of a single was determined by multiplying leaf length, the greatest leaf width and a specific crop coefficient factor of 0.70 [25]. Finally the LAI (m^2^ m^-2^) of maize was determined as the ratio of leaf area to ground area (S1 Fig).

#### Soil water content dynamics

SWC was recorded once a week in the three replicates of maize-soybean relay strip intercropping in 2013 and 2014 seasons. At 0–30 cm soil depth, the SWC was measured gravimetrically, whereas variations in the SWC from 30–90 cm were quantified using neutron probes. The access tubes were allocated to three different locations between two proximate maize and soybean plants in the central row of maize strip (CRM), adjacent row between maize and soybean strip (AR), and central row of soybean strip (CRS). Finally, the mean at each soil depth was taken to measure SWC.

#### Soil evaporation

We used microlysimeters (MLs) to measure soil E in the three replicates of maize-soybean relay strip intercropping. For sole cropping treatments, MLs were positioned in the inter-row of the plants. However, they were located in the central row of maize strip (CRM), adjacent row between maize and soybean strip (AR), and central row of soybean strip (CRS) of maize-soybean intercrop systems. MLs consist of an inner and outer core were constructed using polyvinylchloride (PVC). Inner core had a length of 14 cm, internal diameter of 10 cm and external diameter of 10.5 cm. While outer core made with a length and diameter of 13 cm and 11 cm, respectively. Plastic film was used to seal the base of each inner core in order to avoid the possibility water outflow. The outer core was slowly hammered into the field and the inner core was installed in it. Finally, a portable electronic balance (LP-3102) was used at 8.00 a.m. each day to weight MLs and measure the average daily soil evaporation.

#### Throughfall

Throughfall was estimated from silking to physiological maturity of maize in the three replicates of maize-soybean relay strip intercropping. For this purpose, 42 home-made circular rainwater collectors each with a diameter of 16 cm (the maximum rainwater collection of which 124.4 mm) were located in the central row of maize strip (CRM), adjacent row between maize and soybean strip (AR) and central row of soybean strip (CRS). Measurement time was determined according to the weather conditions, and the weather information was collected from the Renshou Bureau of Meteorology in 2013 and 2014 growing seasons.

#### Grain yield and land equivalent ratio

A 50 m^2^ net area of each plot was selected to calculate the grain and biomass yields of maize and soybean for different treatments in intercropping and sole cropping. In order to minimize the border row effect, the 0.5 m plot area with first two and last two plants were excluded. All the three replicates were taken into account during the measurements and maize and soybean plants from three replicates were harvested. After recording the total fresh weight, the cobs/pods from the stover of maize and soybeans were separated, sun-dried, packed and threshed. A moisture meter was used to determine the moisture content of grains. Finally, the grain yields were recorded when the moisture content of grains dropped to 12%.

$$Grain\;\mathrm{yield}\;(\mathrm{kg}\;m^{-2})=\mathrm{dry}\;\mathrm{weight}\;(\mathrm{kg}\;m^{-2})/\mathrm{net}\;\mathrm{area}\;(m^{2})$$ (1)

Similarly, the LER, which is the land use index and indicates the benefit of yield produced by the maize-soybean intercrops over that of corresponding sole crops, was calculated using the formula [26]:

$$LER={\mathrm{LER}}_{M}+{\mathrm{LER}}_{S}=\frac{Y_{M,I}}{Y_{M,S}}+\frac{Y_{S,I}}{Y_{S,S}}$$ (2)

Where, LER_M_ and LER_S_ represent the partial land equivalent ratio of maize and soybean, respectively. The yield of intercropped and sole maize crops are represented as *Y*_*M*, *I*_ and *Y*_*M*, *S*_ and the yield of intercropped soybean and sole soybean are shown as Y_S,I_ and Y_S, S_, respectively.

#### Water use efficiency and water equivalent ratio

Before measuring WUE, we used the soil water balance equation to estimate the crop evapotranspiration (ET_c_) of maize and soybean in intercropped and sole cropped treatments.

$$ET_{c}=\Delta W+I+R-Q-SI$$ (3)

The experiments were performed under fully rainfed condition in a sub-humid climate and no irrigation was supplied throughout the 2013 and 2014 growing seasons. We therefore used the following equation to estimate the ET_c_ of maize and soybean in intercropped and sole cropped treatments.

$$ET_{c}=\Delta W+R-Q-SI$$ (4)

Where, ΔW shows soil water changes (mm) in the top 2 m soil profile during the whole growth period, R is the total rainfall (mm), Q is the actual runoff (mm) determined by soil conservation service runoff model [27], whereas SI shows the ground water discharge from the root zone (mm), and is given by an expression:

SI=−k(Δh/Δz) (5)

Where, k shows hydraulic conductivity and Δh is the difference in hydraulic potential over the depth interval Δz at the end of the root zone. The exponential relationship between k and θ was considered to estimate the hydraulic conductivity [28]. Soil retention curves were used to calculate the matric potential of soil water [29–30].

Finally, the WUEs of maize and soybean in different treatments of relay strip intercropping and sole cropping systems were determined using the formula [31].

$$WUE=Y/ET_{c}$$ (6)

Where, Y is grain yield (kg ha^-1^), ET_c_ is crop evapotranspiration

Similarly, the group WUE (S1 Table) was computed using the formula:

$$Group\;WUE=Y_{M}+Y_{S}/GETc$$ (7)

Where, Y_M_,Y_S_ are maize and soybean yields, respectively. GET_c_ is the group crop evapotranspiration (S1 Table), which is computed from the changes in soil water content and whole growth period rainfall.

$$GET_{c}<ET_{M}+ET_{S}$$ (8)

To calculate the WERs of different treatments in maize-soybean intercrop systems, we used a formula proposed by [32–33]:

$$WER={\mathrm{WER}}_{M}+{\mathrm{WER}}_{S}=\frac{WUE_{M,I}}{WUE_{M,S}}+\frac{WUE_{S,I}}{WUE_{S,S}}$$ (9)

Where, WER_M_, WER_S_ represent the water equivalent ratio of maize and soybean and WUE_M, I_ WUE_M, S_, WUE_S, I_, WUE_S, S_, represent the relative water use efficiency of intercropped maize, sole maize and sole soybean, respectively. WER shows the water use advantage and define as the relative amount of water that would be required for sole crops to produce yield equivalent to that of corresponding intercrops.

#### Data analysis

Microsoft Excel 2010 and SAS 9.3 were used for data analysis. We performed one-way analysis of variance to test the effects of changes in planting geometries on WUE and ET_c_ of maize-soybean intercrop systems using 160 and 200 cm bandwidth. OriginPro 8 was used to draw figures and determine the determination coefficient (R^2^) values. The comparisons among different treatments were made using Duncan’s Multiple Range test at P ≤ 0.05.

## Results

### Dynamics of maize leaf area index

**Fig 3:**
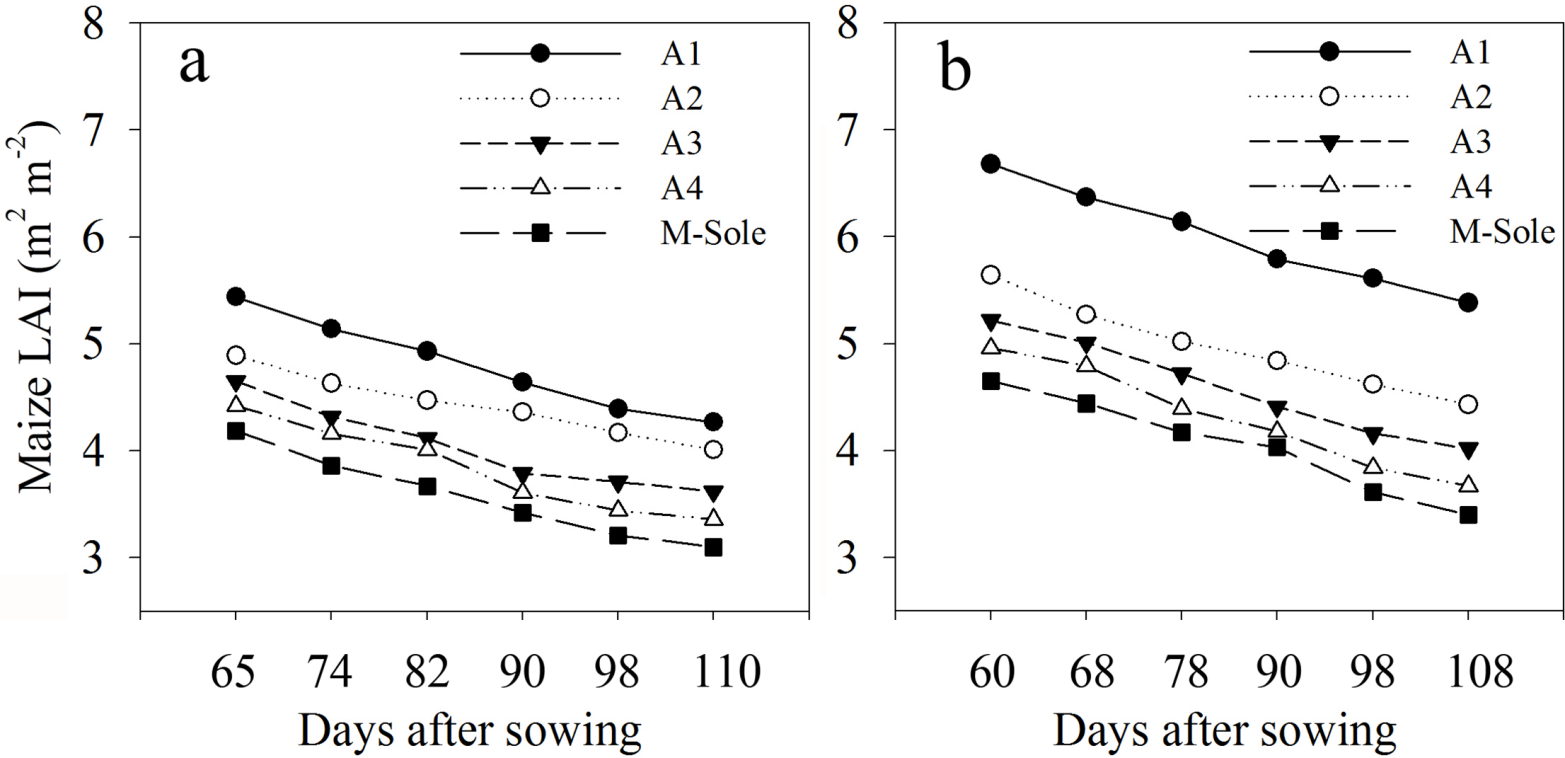
Maize LAI dynamics as affected by planting geometries in 2013 and 2014 seasons. (a) 160 cm bandwidth: A1 = 20:140 cm; A2 = 40:120 cm; A3 = 60:100 cm; A4 = 80:80 cm. (b) 200 cm bandwidth: A1 = 20:180 cm; A2 = 40:160 cm; A3 = 60:140 cm; A4 = 80:120 cm. Each data point represents standard deviation of the mean (n = 3).

LAI of maize showed substantial variations from 60–110 days after sowing (DAS) in the different planting geometries of maize-soybean relay strip intercropping and their sole cropping (Fig 3A and 3B).

**Fig 4:**
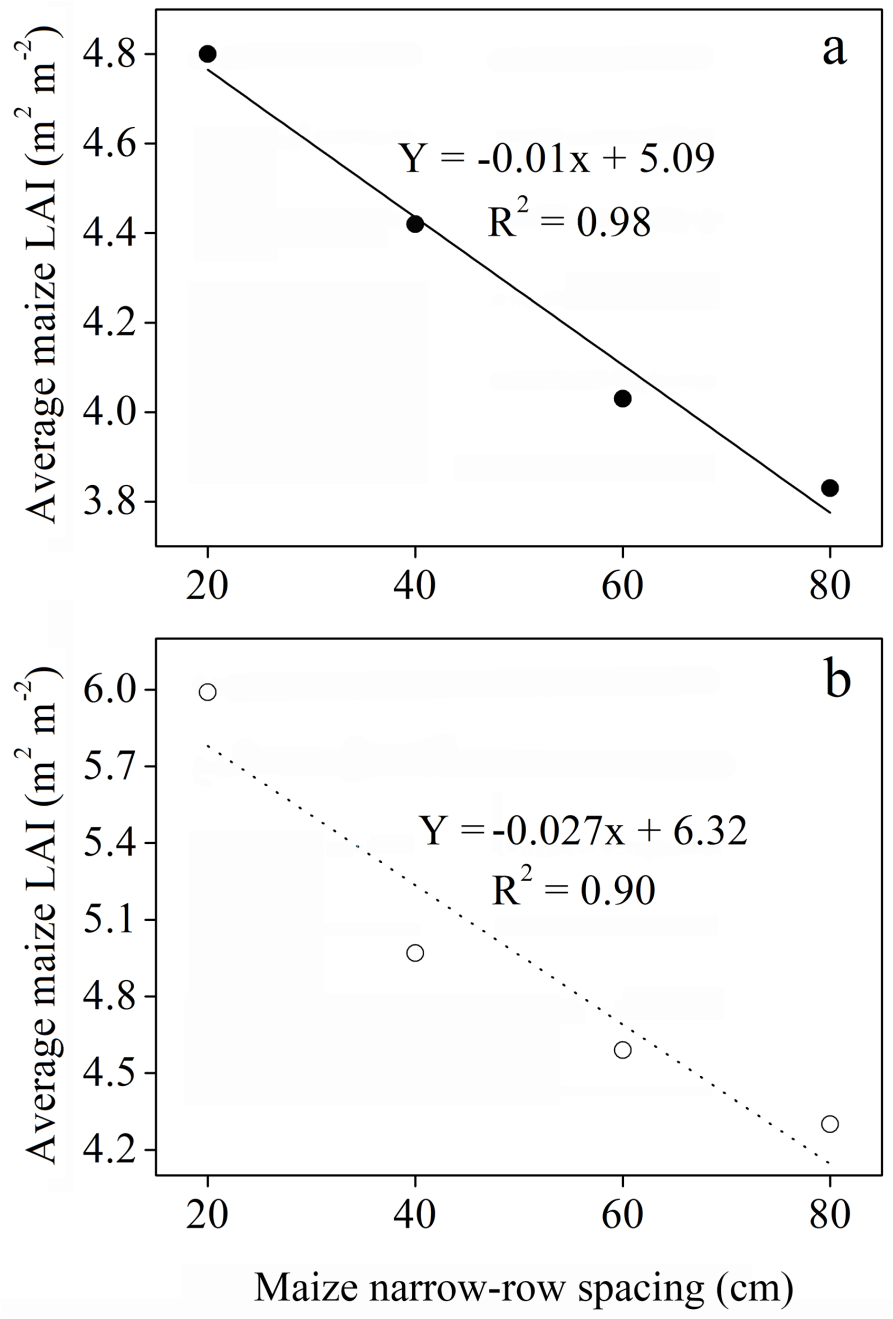
Average maize LAI as a function of maize narrow-row spacing in 2013 and 2014 seasons. Solid line and circles represent values from 2013, whereas dashed line and empty circles represent values from 2014.

The effects of changes in planting geometries on canopy structure and coverage of maize plants were remarkable. Greater LAI was measured for intercropped maize in the different planting geometries compared to sole maize. At 60–65 DAS, the highest maize LAI (5.44 and 6.68 m^2^ m^-2^) was measured for the 20:140 cm and 20:180 cm planting geometries using 160 cm and 200 cm bandwidth, respectively. However, the LAI of intercropped maize gradually declined thereafter and reached a minimum at 108–110 DAS for the 80:80 cm and 80:120 cm planting geometries using 160 cm and 200 cm bandwidth, respectively. The LAI of intercropped maize had shown a similar trend to that of sole maize over two growing seasons. However, the values of LAI for sole maize were relatively lower (never exceeded 4.65 m^2^ m^-2^) compared to those for intercropped maize. Moreover, there was a significantly negative relationship between average maize LAI and maize narrow-row spacing with determination coefficient (R^2^) values of 0.98 and 0.90 at 160 cm and 200 cm bandwidth, respectively (Fig 4A and 4B; P < 0.05).

### Soil water content dynamics

Based on the results, SWC in the different planting geometries of 200 cm bandwidth was relatively lower compared to those of 160 cm bandwidth (Table 1; P < 0.05).

**Table 1:** Soil water content (%) for different planting geometries in 2013 and 2014 seasons.

**Treatment**							
**Bandwidth**	**Place**	**20:140cm**	**40:120cm**	**60:100cm**	**80:80cm**	**M-sole**	**S-sole**
**160 cm**	CRM	20.80b	21.16b	21.36b	21.82b	21.40	—
	AR	21.35b	22.45a	22.40a	23.05a	—	—
	CRS	22.74a	22.77a	22.63a	22.17b	—	21.28
	Mean	21.63bcd	22.12abc	22.13abc	22.35ab	21.40cd	21.28d
	CRM	23.31b	23.49b	24.03a	24.08a	23.31	—
	AR	23.39b	24.42b	23.09b	23.86a	—	—
	CRS	25.00a	24.80a	24.25a	24.08a	—	22.95
	Mean	23.90abc	24.23abc	23.79abc	24.08abc	23.31bc	22.95c
**200 cm**	**Place**	**20:180cm**	**40:160cm**	**60:140cm**	**80:120cm**	**M-sole**	**S-sole**
	CRM	12.18c	12.66c	12.78b	13.56a	13.06	—
	AR	13.38b	13.63b	12.65b	12.73b	—	—
	CRS	14.10a	14.46a	14.87a	13.59a	—	12.37
	Mean	13.22cd	13.58b	13.44bc	13.29cd	13.06de	12.37f
	CRM	17.97c	19.57b	19.78c	20.68a	20.30	—
	AR	18.76b	19.42b	20.54b	19.80b	—	—
	CRS	19.29a	21.03a	21.24a	19.73b	—	18.12
	Mean	18.67d	20.01b	20.52a	20.07e	20.30ab	18.12e

CRM = central row of maize strip, AR = adjacent row between maize-to-soybean strip, CRS = central row of soybean strip. M-sole = sole Maize, S-sole = sole soybean.

Means in columns followed by the same lower case letters are not significantly different using Duncan’s Multiple Range test (P < 0.05; n = 3).

**Fig 5:**
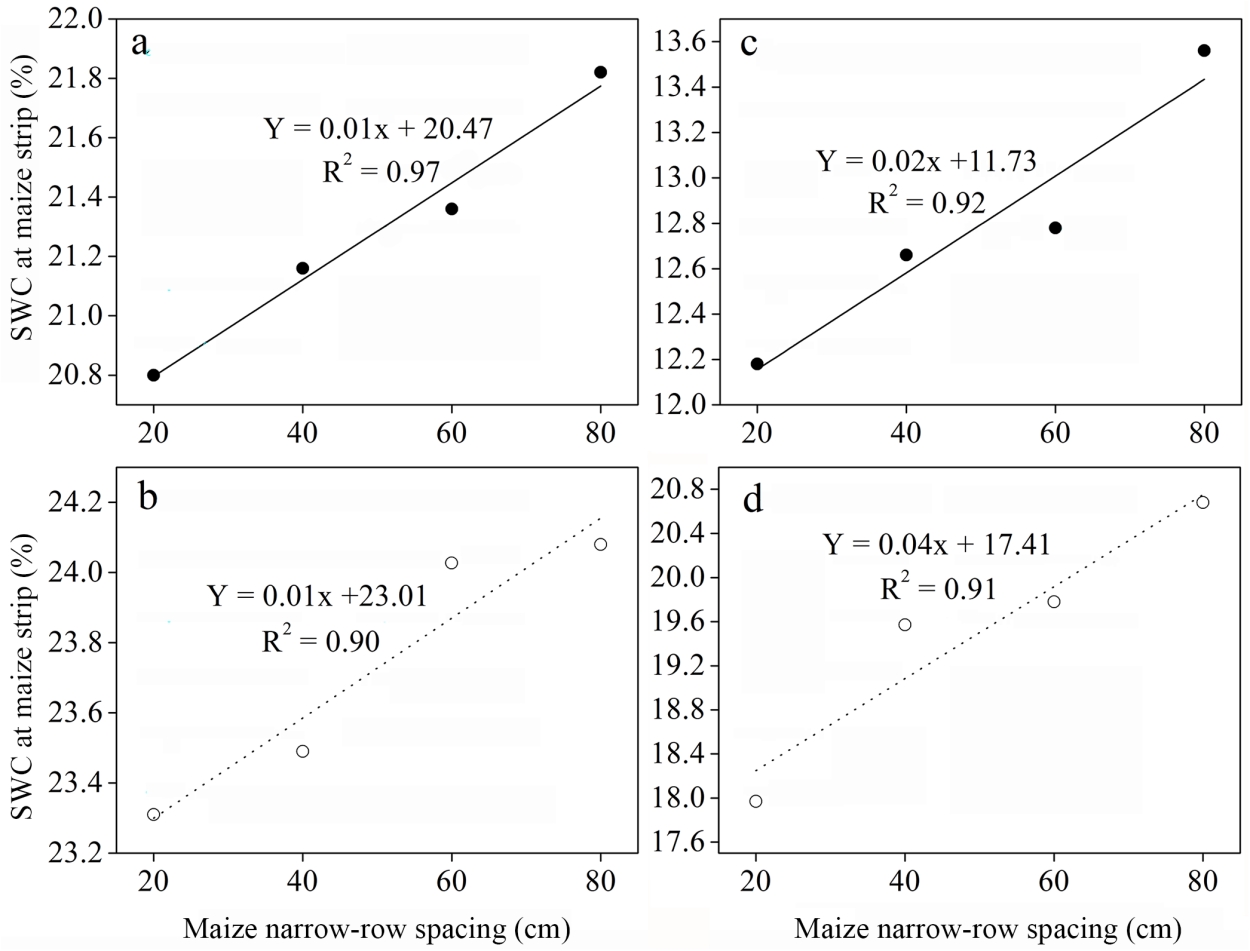
Soil water content as a function of maize narrow-row spacing in 2013 and 2014 seasons. (a, b) 160 cm bandwidth and (c, d) 200 cm bandwidth

Besides, SWC in the central row of maize strip was considerably lower than those in the adjacent row between maize and soybean strip and central row of soybean strip: central row of maize (CRM) < adjacent row between maize and soybean (AR) < central row of soybean (CRS). On overall basis, the average SWC in the different planting geometries of intercropping was relatively greater compared to sole cropping. At 160 cm bandwidth, the highest average SWC (22.35% and 24.23%) was recorded for the 80:80 cm and 20:140 cm planting geometries, respectively. In contrast, when we increased the bandwidth to 200 cm, the highest SWC (13.58% and 20.52%) was appeared for the 40:160 cm and 60:140 cm planting geometries, respectively. Moreover, there was a significantly positive relationship between SWC of maize strip and maize narrow-row spacing with determination coefficient (R^2^) values of 0.97 and 0.90 at 160 cm bandwidth and 0.92 and 0.91 at 200 cm bandwidth (Fig 5A–5D; P < 0.05).

### Soil evaporation

The average daily soil E measured by microlysimeters over the growing seasons of 2013 and 2014 is presented in Table 2.

**Table 2:** Soil evaporation (mm day^-1^) for different planting geometries in 2013 and 2014 seasons.

**Treatment**							
**Bandwidth**	**Place**	**20:140cm**	**40:120cm**	**60:100cm**	**80:80cm**	**M-sole**	**S-sole**
**160 cm**	CRM	1.75c	1.83b	1.84b	1.86a	1.98	—
	AR	2.0b	2.06ab	1.94ab	1.85b	—	—
	CRS	2.39a	2.16a	2.04a	1.94a	—	2.91
	Mean	2.05b	2.01b	1.94b	1.88b	1.98b	2.91a
**200 cm**	**Place**	**20:180cm**	**40:160cm**	**60:140cm**	**80:120cm**	**M-sole**	**S-sole**
	CRM	2.06b	2.14b	2.16b	2.19b	2.05	—
	AR	2.73a	2.07b	2.46a	2.22a	—	—
	CRS	2.87a	2.49a	2.35a	2.31a	—	3.03
	Mean	2.56b	2.23de	2.32cd	2.24de	2.05e	3.03a

CRM = central row of maize strip, AR = adjacent row between maize-to-soybean strip, CRS = central row of soybean strip. M-sole = sole maize, S-sole = sole soybean.

Means in columns followed by the same lower case letters are not significantly different using Duncan’s Multiple Range test (P < 0.05; n = 3).

**Fig 6:**
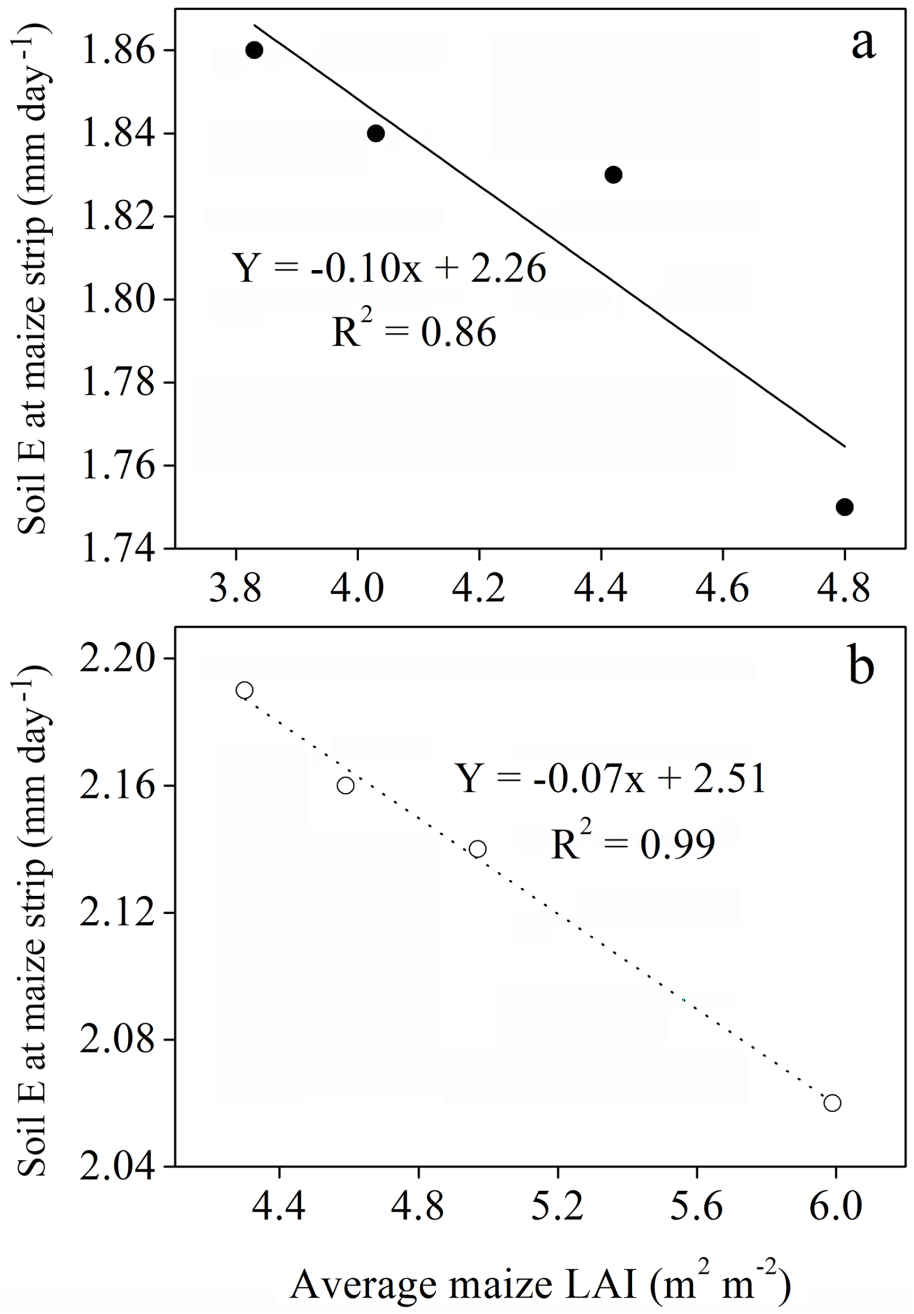
Soil evaporation as a function of average maize LAI in 2013 and 2014 seasons. (a) 160 cm bandwidth and (b) 200 cm bandwidth. Solid line and circles represent values from 2013, whereas dashed line and empty circles represent values from 2014.

In general, soil E in different treatments of 160 cm bandwidth was lower compared to those of 200 cm bandwidth. A decreasing trend of soil E was observed within the three strips of intercropping in the following order: central row of maize strip (CRM) < adjacent row between maize and soybean strip (AR) < central row of soybean strip (CRS). With increasing distance between maize narrow-row spacing from 20 cm to 80 cm, the soil E of maize strip was gradually increased. Contrasting opposite trend was found for the soil E of soybean strip, whereas soil E of maize-to-soybean strip changed inconsistently. Compared to sole soybean, the soil E was remarkably reduced by both 160 cm and 200 cm bandwidth under maize-soybean intercropping, however, this was slightly higher than that of sole maize. The lowest average soil E values were 1.88 mm day^-1^ for the 80:80 cm planting geometry and 2.23 mm day^-1^ for the 40:160 planting geometry using 160 cm and 200 cm bandwidth, respectively. Furthermore, the determination coefficient (R^2^) values of 0.86 at 160 cm bandwidth and 0.99 at 200 cm bandwidth, indicating a significantly negative relationship between soil E and average maize LAI (Fig 6A and 6B; P < 0.05).

### Throughfall

Throughfall variations for different treatments of maize-soybean relay strip intercropping and their sole cropping over two years are shown in Table 3.

**Table 3:** Throughfall (mm) for different planting geometries in 2013 and 2014 seasons.

**Treatment**							
**Bandwidth**	**Place**	**20:140cm**	**40:120cm**	**60:100cm**	**80:80cm**	**M-sole**	**S-sole**
**160 cm**	CRM	0.08c	0.41c	1.11c	1.90a	1.14	—
	AR	2.57b	1.82b	2.46b	1.96a	—	—
	CRS	4.63a	5.62a	4.38a	2.59a	—	3.60
	Mean	2.43cd	2.62b	2.65b	2.15d	1.14e	3.60a
	CRM	7.09c	21.25b	26.30b	29.31a	30.08	—
	AR	17.62b	24.80b	22.49b	18.78a	—	—
	CRS	52.82a	63.20a	42.07a	34.34a	—	44.91
	Mean	25.84de	36.42b	30.29c	27.47d	30.08c	44.91a
**200 cm**	**Place**	**20:180cm**	**40:160cm**	**60:140cm**	**80:120cm**	**M-sole**	**S-sole**
	CRM	10.45b	23.39b	26.14b	34.21b	35.46	—
	AR	16.42b	40.04b	27.42b	27.62b	—	—
	CRS	77.70a	82.44a	89.55a	95.62a	—	75.87
	Mean	34.86d	48.62c	47.70c	52.58b	35.46d	75.87a
	CRM	13.44b	21.65b	25.98b	36.38b	37.93	—
	AR	15.84b	29.94b	32.18b	18.81c	—	—
	CRS	88.82a	69.72a	90.43a	80.36a	—	85.66
	Mean	39.37bc	40.43bc	49.53b	34.52d	37.93cd	85.66a

CRM = central row of maize strip, AR = adjacent row between maize-to-soybean strip, CRS = central row of soybean strip. M-sole = sole Maize, S-sole = sole soybean.

Means in columns followed by the same lower case letters are not significantly different using Duncan’s Multiple Range test (P < 0.05; n = 3).

**Fig 7:**
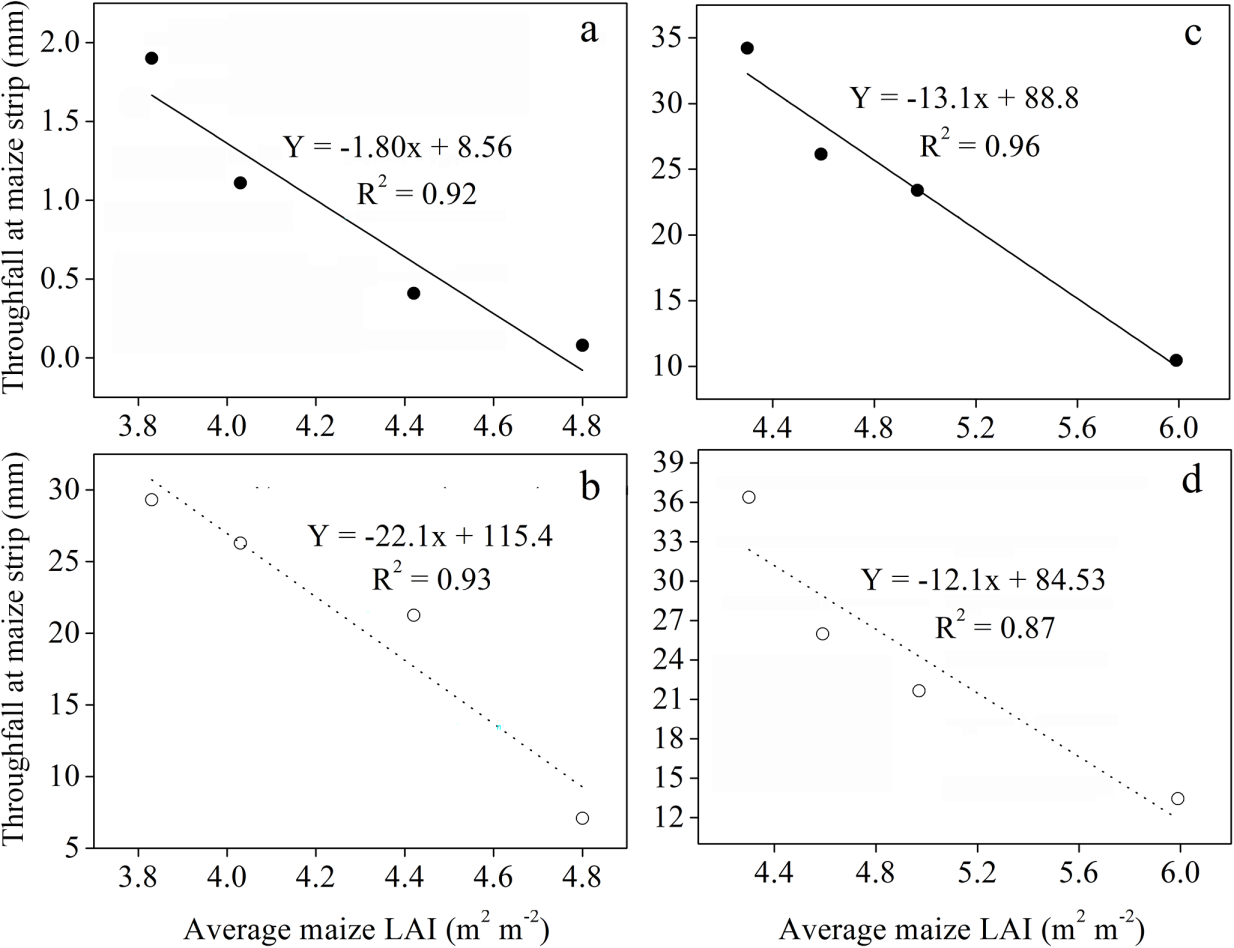
Throughfall as a function of average maize LAI in 2013 and 2014 seasons. (a, b) represent values at 160 cm bandwidth and (c, d) represent values at 200 cm bandwidth.

Throughfall was found to vary pronouncedly within the three strips of intercropping and decreased with changes in planting geometries in the following order: central row of maize strip (CRM) < adjacent row between maize and soybean strip (AR) < central row of soybean strip (CRS). At 200 cm bandwidth, throughfall was reletively higher compared to that of 160 cm bandwidth. Throughfall for maize strip was significantly increased with increasing distance between maize narrow-row spacing, however, there were no consistent variations seen in throughfall for soybean strip and maize-to-soybean strip. On overall basis, higher throughfall was recorded for sole soybean when compared with sole maize and intercropped treatments. In addition, there was a significant negative relationship between throughfall of maize strip and average maize LAI with determination coefficient (R^2^) values of 0.92 and 0.93 at 160 cm bandwidth and 0.96 and 0.87 at 200 cm bandwidth (Fig 7A–7D; P < 0.05).

### Grain yield and land equivalent ratio

Grain yields and LERs in the different treatments of maize-soybean intercropping and their sole cropping are shown in Table 4.

**Table 4:** Grain yields and LERs for different planting geometries in 2013 and 2014 seasons.

**160 cm bandwidth**				
**Treatment**	**Maize yield (kg ha^-1^)**	**Soybean yield (kg ha^-1^)**	**Total yield (kg ha^-1^)**	**LER**
20:140cm	4906.75c	1319.10b	6225.85c	1.41b
40:120cm	6826.74b	1238.73b	8065.46a	1.62a
60:100cm	6609.99bc	1167.08b	7777.06ab	1.55ab
80:80cm	6600.98bc	1117.53b	7718.51ab	1.52ab
M-sole	7387.58a	—	7387.58b	—
S-sole	—	1791.99a	1791.99d	—
**200 cm bandwidth**				
20:180cm	9037.2d	1850.9b	10888.1d	1.73b
40:160cm	10282.4b	1733.4c	12015.8a	1.79a
60:140cm	10183.9bc	1565.8d	11749.7b	1.70b
80:120cm	9677.5c	1485.7d	11163.2c	1.61c
M-sole	10868.6a	—	10868.6d	—
S-sole	—	2061a	2061e	—

Means in columns followed by the same lower case letters are not significantly different using Duncan’s Multiple Range test (P < 0.05; n = 3).

The results exhibited that maize-soybean intercropping has a total yield advantage. The grain yields of maize and soybean intercrops were slightly lower than those of sole crops. However, the total yields of maize-soybean intercrop systems were significantly higher than those of sole cropping. In 2 years, the grain yields of intercropped maize was significantly increased with increasing distance between maize narrow-row spacing from 20 cm to 40 cm, however, gradually declined thereafter. Contrasting an opposite trend was found for the grain yields of intercropped soybean. The highest grain yields of sole maize were 7387.58 and 10868.6 kg ha^-1^ and the highest grain yields of sole soybeans were 1791.99 and 2061 kg ha^-1^ in 2013 and 2014 seasons, respectively. While the highest total yields of 8065.46 kg ha^-1^ and 12015.8 kg ha^-1^ were recorded for the 40:120 cm and 40:160 cm planting geometries using 160 cm and 200 cm bandwidth, respectively. Meanwhile, LERs in the different planting geometries of intercropping varied from 1.41 to 1.62 at 160 cm bandwidth and 1.61 to 1.79 at 200 cm bandwidth (Table 4). The highest LERs of 1.62 for the 40:120 cm planting geometry and 1.79 for the 40:160 cm planting geometry, indicating that maize-soybean relay strip intercropping has a land use advantage.

### Water use efficiency and water equivalent ratio

The actual ET_c_ of sole maize was 283.5 mm and 309.4 mm and sole soybean was 269.47 mm and 297.50 mm. The results exhibited that changes in planting geometries did not significantly reduce water consumption (ET_c_) of maize-soybean intercrops (Table 5; *P* < 0.05).

**Table 5:** ETc, WUEs, Gwue and WERs for different planting geometries in 2013 and 2014 seasons.

**160 cm bandwidth**						
**Treatment**	**MET_c_**	**SET_c_**	**Mwue**	**Swue**	**Gwue**	**WER**
	**(mm)**	**(mm)**	**(kg ha^-1^ mm^-1^)**	**(kg ha^-1^ mm^-1^)**	**(kg ha^-1^ mm^-1^)**	****
20:140cm	290.5a	274.6a	16.89c	4.80b	17.80c	1.45c
40:120cm	286.5a	273.5a	23.83b	4.53b	23.06b	1.66a
60:100cm	284.4a	271.9a	23.24bc	4.29c	22.23bc	1.51b
80:80cm	284.6a	271.7a	23.19bc	4.11c	22.06bc	1.54b
M-sole	283.5a	—	26.05a	—	26.05d	—
S-sole	—	269.5a	—	6.65a	6.65d	—
**200 cm bandwidth**						
20:180cm	316.3a	301.5a	28.58d	6.14b	23.74c	1.70b
40:160cm	314.5a	300.0a	32.69b	5.78c	26.21b	1.76a
60:140cm	312.4a	298.7a	32.60b	5 24d	25.62c	1.69b
80:120cm	311.5a	299.0a	31.07c	4.97d	24.34d	1.60c
M-sole	309.4a	—	35.12a	—	35.12a	—
S-sole	—	297.5a	—	6.93a	6.93f	—

MET_c_ = maize evapotranspiration, SET_c_ = soybean evapotranspiration, Mwue = maize water use efficiency, Swue = soybean water use efficiency, Gwue = group water use efficiency, WER = water equivalent ratio. Means in columns followed by the same lower case letters are not significantly different using Duncan’s Multiple Range test (P < 0.05; n = 3).

The WUE of intercropped maize increased with increasing distance between maize narrow-row spacing from 20 to 40 cm, however, declined thereafter during both seasons. In contrast, the WUE of intercropped soybean decreased with increasing the distance between narrow maize rows from 20 to 80 cm. The WUEs of maize-soybean intercrops were comparatively lower than those of sole crops. However, the group WUE of maize-soybean intercrop systems was significantly greater than that of sole cropping. An increase in group WUE was observed with increasing distance between narrow maize rows from 20 to 40 cm, which disappeared thereafter. The highest group WUEs of 23.06 and 26.21 kg ha^-1^ mm^-1^ for the 40:120 cm and 40:160 cm planting geometries, respectively, were 39% and 23% higher than those for sole cropping. Furthermore, the WER values varied from 1.45 to 1.66 at 160 cm bandwidth and 1.60 to 1.76 at 200 cm bandwidth (Table 5). The highest WER values of 1.66 for the 40:120 cm planting geometry and 1.76 for the 40:160 cm planting geometry, indicating the water use advantage of maize-soybean intercropping.

## Discussion

Intercropping involves the growing of two or more crop species different in their growth habit, phenological attributes, resource use, and productivity [34]. Maize-soybean intercrops have shown to use resources in different spatial and temporal sequences owing to their root length density and canopy structure [35]. Optimum planting geometry could increase the total yields for maize-soybean relay strip intercrop systems [13]. In this study, we investigated the effects of changes in planting geometry on WUE and ET_c_ of maize and soybean intercrops. During the two-year period, the LAI of maize in the different planting geometries of intercropping using 160 and 200 cm bandwidth was considerably higher compared to that of sole maize (Fig 3A and 3B), which could be due to the border row effect [22, 36]. At 60–65 DAS, the LAI of intercropped maize reached a maximum for the 20:140 cm and 20:180 cm planting geometries. Later LAI of intercropped maize gradually decreased with increasing distance between maize narrow-row spacing. We believe this is a result of decrease in crop coverage and dying of lower leaves [29]. Besides, the negative relationship between average maize LAI and maize narrow-row spacing, implying a reduction in crop coverage with increasing distance between maize narrow-row spacing (Fig 4A and 4B). This result is supported by a previous study, where LAI of maize and LAI affecting photosynthesis (LAIp) were markedly decreased by 4.7–31.2% when the distance between maize narrow-row spacing increased from 35 to 100 cm [37]. In general, the average SWC in different planting geometries of intercropping was greater compared to sole cropping (Table 1). This result is synonymously related with those from previously reported studies, demonstrating that when roots are in drying soil, substantial amount of abscisic acid (ABA) can be produced and transported through the xylem to the shoots, thereby regulating stomatal opening and thus helping in soil water saving [38, 39, 40]. Furthermore, the imbalanced but slightly higher SWC within the three strips of maize-soybean relay strip intercrop systems may be due to the difference in root mass density and crop coverage of maize and soybean, as each component crop in strip intercrop systems more likely taken up the water from its strip first and intermixed zone later [29, 41]. Similarly, changes in planting geometries had decreased the canopy coverage of maize plants, thereby altering the microclimate of the field by increasing air transport, decreasing air water vapor content and ultimately resulting in greater soil E [42]. In addition, a decreasing trend of soil E within the three strips of intercropping was more likely due to the difference in canopy coverage and root mass density of maize and soybean (Table 2). This result is in agreement with those from previously published reports [29, 41]. Greater soil E in different planting geometries of intercropping compared to sole maize was closely related with poor covering of the surface soil by the wide soybean rows, whereas on the other side, sole maize provided a uniform covering to the surface soil and resulting in lower soil E [43]. Throughfall had shown remarkable variations within the three strips of intercropping, however, throughfall of maize strip showed a notable increment with increasing distance between maize narrow-row spacing (Table 3). This was rather because of the changes in canopy structure of maize plants related to planting geometries [29, 44]. Despite of changes in canopy structure, higher average rainfall in 2014 than that in 2013 could probably also accounted for the higher throughfall of the 200 cm bandwidth (Fig 1). Moreover, the lower grain yields of maize-soybean intercrops compared to corresponding sole crop are consistent with those from previously published reports [16, 45]. The narrow maize row spacing had a dominant effect on the grain yields of intercropped maize. The lowest grain yields of intercropped maize for the 20:140 cm and 20:180 cm planting geometries could be associated with the severe intraspecific competition between maize plants. However, the intraspecific competition between maize plants weakened and the grain yields of intercropped maize reached a maximum with increasing distance between maize narrow-row spacing. Contrasting a decline was seen for the grain yields of intercropped soybean with increasing distance between maize narrow-row spacing (Table 4). This was more likely due to the shading effect caused by maize plants on soybeans in the relay strip intercrop systems [13, 46]. The highest total yields for the 40:120 cm planting geometry using 160 cm bandwidth and 40:160 cm planting geometry using 200 cm bandwidth could be due to improvement in the light environment of the soybean canopy and lower intraspecific competition [13]. The variations in LERs ranged from 1.41–1.62 in 2013 and 1.61–1.79 in 2014, indicating that planting geometries directly affect LERs (Table 4). Moreover, the lower WUEs of maize and soybean intercrops than those of sole crops could be due to the lower grain yields and longer growth period of relay strip intercrop systems (Table 5). This result is in line with those from previously published reports [47–48]. However, it is noteworthy that the highest group WUEs of 23.06 and 26.21 kg ha^-1^ mm^-1^ for the 40:120 and 40:160 cm planting geometries, respectively, were 39% and 23% higher than those for sole cropping (S1 Table). In addition, the highest WERs 1.66 for the 40:120 cm planting geometry and 1.76 for the 40:160 cm planting geometry could possibly due to the complimentary root distribution of maize and soybean intercrops, which led to a significant water use advantage [49].

## Conclusions

The opportunities for increasing the effective water use through intercropping are limited. Our results confirm that increasing the distance between maize narrow-row spacing reduced the canopy coverage and LAI for intercropped maize, thereby decreasing SWC, soil E, and throughfall in the following order: central row of maize strip (CRM) < adjacent row between maize and soybean strip (AR) < central row of soybean strip (CRS). Compared to sole soybean, the soil evaporative water loss in maize-soybean relay strip intercrop systems was greatly minimized by the 40:160 cm planting geometry and 200 cm bandwidth. Moreover, the highest total yields and LERs for the 40:160 cm planting geometry using 200 cm bandwidth, indicating the yield and land use advantage, respectively. The lower WUEs of intercrops were mainly due to their lower grain yields compared to corresponding sole crops. However, the highest group WUEs and WERs for the 40:160 cm planting geometry using 200 cm bandwidth, suggesting that an optimum planting geometry of 40:160 cm and bandwidth of 200 cm could be a viable planting pattern management method for attaining high group WUE in maize-soybean relay strip intercrop systems. Further studies under limited water conditions may be useful to explore greater gains in the group WUE of maize-soybean relay strip intercrop systems.

## Supporting information

S1 Fig LAI dynamics of maize for the different planting geometries. (XLSX)

S1 Table Calculation for the Gwue of different treatments. (XLSX)
